# Survival trends for left and right sided colon cancer using population‐based SEER database: A forty‐five‐year analysis from 1975 to 2019

**DOI:** 10.1002/cam4.7145

**Published:** 2024-04-23

**Authors:** Mark B. Ulanja, Kwabena Oppong Asafo‐Agyei, Vijay Neelam, Bryce D. Beutler, Daniel Antwi‐Amoabeng, Samuel B. Governor, Ganiyu A. Rahman, Francis T. Djankpa, Reginald N. Ulanja, Grace B. Nteim, Tarig Mabrouk, Millicent Amankwah, Olatunji B. Alese

**Affiliations:** ^1^ CHRISTUS Ochsner St. Patrick Hospital Lake Charles Louisiana USA; ^2^ CHRISTUS Highland Medical Center Shreveport Louisiana USA; ^3^ Department of Radiology, Keck School of Medicine University of Southern California Los Angeles California USA; ^4^ Saint Louis University College for Public Health and Social Justice Saint Louis Missouri USA; ^5^ Department of Surgery, School of Medical Sciences University of Cape Coast Cape Coast Ghana; ^6^ Department of Physiology, School of Medical Sciences University of Cape Coast Cape Coast Ghana; ^7^ Department of Hematology Oncology, Feist‐Weiller Cancer Center Louisiana State University Health Shreveport Louisiana USA; ^8^ Department of Hematology and Oncology Winship Cancer Institute, Emory University Atlanta Georgia USA

**Keywords:** cause‐specific survival, competing risk analysis, left sided colon cancer, overall survival, right sided colon cancer, SEER database, survival trends

## Abstract

**Background:**

Survival differences between left‐sided colon cancer (LSCC) and right‐sided colon cancer (RSCC) has been previously reported with mixed results, with various study periods not accounting for other causes of mortality.

**Purpose:**

We sought to assess the trends in colon cancer cause‐ specific survival (CSS) and overall survival (OS) based on sidedness.

**Method:**

Fine‐Gray competing risk and Cox models were used to analyze Surveillance, Epidemiology, and End Results (SEER) population‐based cohort from 1975 to 2019. Various interval periods were identified based on the timeline of clinical adoption of modern chemotherapy (1975–1989, interval period A; 1990–2004, B; and 2005–2019, C).

**Results:**

Of the 227,637 patients, 50.1% were female and 46.2% were RSCC. RSCC was more common for African Americans (51.5%), older patients (age ≥65; 51.4%), females (50.4%), while LSCC was more common among Whites (53.1%; *p* < 0.001), younger patients (age 18–49, 64.6%; 50–64, 62.3%; *p* < 0.001), males (58.1%; *p* < 0.001). The Median CSS for LSCC and RCC were 19.3 and 16.7 years respectively for interval period A (1975–1989). Median CSS for interval periods B and C were not reached (more than half of the cohort was still living at the end of the follow‐up period). Adjusted CSS was superior for LSCC versus RSCC for the most recent interval period C (HR 0.89; 0.86–0.92; *p* < 0.001). LSCC consistently showed superior OS for all study periods. Stage stratification showed worse CSS for localized and regional LSCC in the earlier study periods, but the risk attenuated over time. However, left sided distant disease had superior CSS per stage for all interval periods. OS was better for LSCC irrespective of stage, with gradual improvement over time.

**Conclusion:**

LSCC was associated with superior survival compared to right sided tumors. With the adoption of modern chemotherapy regimens, prognosis between LSCC and RSCC became more divergent in favor of LSCC. Colon cancer clinical trials should strongly consider tumor sidedness as an enrollment factor.

## 1 INTRODUCTION

Cancer continues to be the second leading cause of death in the United States. A good proportion was due to colorectal cancer (CRC), accounting for approximately 51,000 of the 602,350 cancer‐related deaths in 2022.^1^ Although treatment strategies continue to advance, the role of colon tumor sidedness is still evolving. Historically, histopathology, molecular characteristics, and pattern of CRC spread were the key elements in the choice of treatment plans and mortality rates. Earlier prognostic studies^2^, ^3^ showed biological variations between proximal (genetically more stable) and distal tumor locations. Other studies showed that right‐sided colon cancers (RSCC) are more likely to be advanced, high grade tumors, while left‐sided colon cancers (LSCC) are more likely to present at early stages with low grade tumors.^4^, ^5^, ^6^

A population‐based comparison study by Benedix et al^7^ showed epidemiological differences, with RSCC diagnosis in significantly older patients, female predominance and associated with higher mortality rates. Some other studies indicated phenotypic variations, with polypoid tumors on the left side and flat type lesions on the right side (*p* < 0.05).^8^ A retrospective survival analysis using the Surveillance, Epidemiology, and End Results (SEER) database that was published in 2008 showed statistically significant differences in median survival rates between RSCC and LSCC (78 vs. 89 months).^9^ Subsequent studies demonstrated worse prognosis with RSCC, regarding overall and cancer specific survival.^10^, ^11^ Other studies; however, showed conflicting results, with varying mortality outcomes depending on stage; prognosis appeared better with RSCC in stages I and II compared to LSCC but conversely for advanced staged tumors.^12^, ^13^

Tumor location may be related to the interplay between immunologic, molecular, and microbiological factors. Differences in survival may exist irrespective of modern therapeutic agents, with an unclear role of tumor sidedness on prognosis. Many of the publications on sidedness used relatively short study periods to examine the impact of CRC sidedness on survival,^2^, ^7^, ^8^, ^9^ without accounting for temporal trends. It is important to assess the natural history of colon cancer sidedness,^14^ and to further examine if survival based on sidedness is related to the adoption of treatment options with increasing efficacy. Furthermore, when analyzing sidedness as a prognostic feature, most studies did not consider “other causes of death” in these patients, known as other cause‐specific survival (OCSS)‐ cause of death unrelated to condition of interest (CRC). We sought to assess the trends in colon cancer cause‐specific survival (CSS)^15^ based on sidedness over a 45‐year period.

## 2 MATERIALS AND METHODS

### 2.1 Study design and population

**FIGURE 1:**
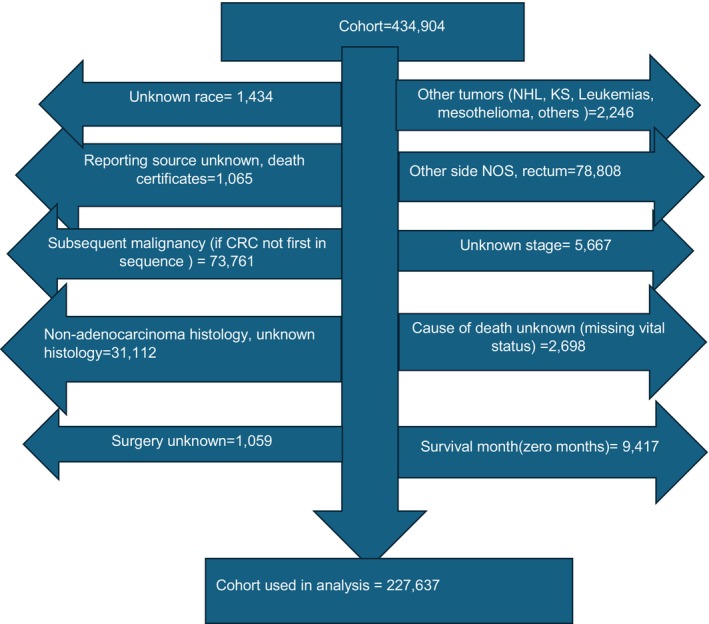
Exclusion criteria. Cases that were excluded are summarized as follows: (1) missing race and/or sex; (2) unknown reporting source; (3) data obtained from death certificates only; (4) missing vital status (dead or alive); (5) missing data related to surgery or surgical resection of the primary neoplasm; (6) survival months equal to zero or not reported; and (7) colon cancer diagnosed as a metachronous, synchronous, or second, third, or subsequent primary neoplasm.

We used the SEER (SEER 8, previously 9*) population‐based cohort from 1975 to 2019 with histologically confirmed CRC diagnosis. The geographical areas covered within the SEER 8 registery include Connecticut, Atlanta, San Francisco‐Oakland, Hawaii, Iowa, New Mexico, Seattle‐Puget Sound, and Utah. The SEER 8 registry was selected as it includes the longest time period among all of the SEER registeries with relevant clinical data dating back to 1975. Various interval periods were identified based on the timeline of clinical adoption of modern chemotherapy (1975–1989, interval period A; 1990–2004, B; and 2005–2019, C).^16^, ^17^, ^18^ The adoption of chemotherapy was based upon National Comprehensive Cancer Network (NCCN) guidelines, which are generally consistent with World Health Organization (WHO) recommendations. Cases were included based on diagnosis using International Classification of Diseases (ICD), Oncology 3rd Edition (ICD‐O‐3) guidelines. Analysis included patients 18–90 years old. Race was categorized as non‐hispanic Whites (NHW), Black, and Asian, Pacific Islander (API)/Alaska Natives (AN). Cases that were excluded are summarized as follows: (1) missing race and/or sex; (2) unknown reporting source; (3) data obtained from death certificates only; (4) missing vital status (dead or alive); (5) missing data related to surgery or surgical resection of the primary neoplasm; (6) survival months equal to zero or not reported, and (7) colon cancer diagnosed as a metachronous, synchronous, or second, third, or subsequent neoplasm. Exclusion criteria are illustrated in Figure 1.

### 2.2 Morphology, sidedness, and staging

All colon cancers with histologic diagnosis based on ICD‐O‐3, and only adenocarcinomas were included in the analysis. The SEER histologic code 8140 was used to identify the cases. Other cases with diagnosis; cystic, mucinous, serous, mucoepidermoid, acinar cell, complex epithelial, epithelia, squamous cell cancers were excluded. Primary rectal adenocarcinomas were also excluded from the analysis. Sidedness was categorized into binary variable as right and left sides. The right sidedness with its primary site label was as follows: cecum (C18.0), ascending colon (C18.2), hepatic flexure (C18.3), and transverse colon (C18.4), while left sidedness as; splenic flexure (C18.5), descending colon (C18.6), sigmoid colon (C18.7), and rectosigmoid junction (C19.9). Staging was grouped using SEER historic staging. This was selected because the American Joint Committee on Cancer (AJCC) staging resulted in high percentage of missing data. The SEER staging was grouped into; localized (limited to site of origin), regional (direct extension and/or regional lymph node(s) involved), and distant (metastatic). Patients with unknown or unstaged SEER historic grouping were excluded from the analysis.

### 2.3 Statistical analysis

The overall survival (OS) and cause‐specific or cancer‐specific survival (CSS) were calculated among patients diagnosed between 1975 and 2019, from the time of diagnosis to time of death or censoring, which ever came first. The other cause of mortality (OCM) was more common as an outcome in comparison to CSS when patients present with early‐stage diseases. As a result, OCM represents a significant competing event for CSS. We therefore used Fine and Gray^19^ sub‐distribution hazard, which is more accurate in estimating covariate's effects on the outcome of interest on absolute scale.^15^ This approach does not censor OCM but treats it as a competing event along CSS in multivariable analysis. Cumulative incidence functions (CIF) which is nonparametric estimates of the cause‐specific survival were calculated, as detailed by Lambert.^20^ The Cox proportional hazard analysis was used to calculate the OS rates in both unadjusted and adjusted models, with vital status as either alive or dead. The variables adjusted for in the adjusted model include the following: age, sex, race, historical stage, surgery, and tumor grade. The analysis was performed using STATA version 16.1 (Stata Corp, College Station, TX). *p* value <0.05 was considered statistically significant.

## 3 RESULTS

### 3.1 Patient characteristics

Of the 227,637 identified colon cancer patients, 50.1% were female and 46.2% (105,091/227,637) were RSCC. There was a relatively even distribution of cases across each time period (1975–1989: 32.0% [72,909/227,637]; 1990–2004: 35.2% [80,024/227,637]; 2005–2019: 32.8% [74,704/227,637]). Racial distribution of LSCC versus RSCC as follows; Black–48.5% versus 51.5%, White–53.1% versus 46.9%, API/AN–63.7% versus 36.3%; *p* < 0.001. Age ≥65 were more likely to have RSCC versus LSCC (51.4% vs. 48.6%), while LSCC was more common for age 18–49 (64.6% vs. 35.4%), 50–64 (62.3% vs. 37.7%), *p* < 0.001 for all. Females were more likely to have RSCC (50.4% vs. 41.9%; *p* < 0.001), while males were more likely to have LSCC (58.1% vs. 49.6%; *p* < 0.001). The rest of the patient's characteristics are shown in Table 1.

**TABLE 1:** Patient characteristics for the entire cohort.

Variable	RSCC			LSCC		
	A = 1975–1989, *n* (%)	B = 1990–2004, *n* (%)	C = 2005–2019, *n* (%)	A = 1975–1989, *n* (%)	B = 1990–2004, *n* (%)	C = 2005–2019, *n* (%)
Median age (IQR)	71 (18–84)	72 (17–84)	69 (17–84)	67 (18–84)	68 (17–84)	62 (18–84)
Age						
18–49	1620 (5.5)	2259 (6.1)	2881 (7.5)	2760 (6.4)	4043 (9.4)	5537 (15.2)
50–64	6526 (22.1)	7598 (20.4)	10,366 (27.0)	13,473 (31.1)	12,384 (28.9)	14,567 (40.1)
65–90	21,398 (72.4)	27,319 (73.5)	25,124 (65.5)	27,132 (62.6)	26,421 (61.7)	16,229 (44.7)
Sex						
Female	16,426 (55.6)	20,375 (54.8)	20,626 (53.7)	20,566 (47.4)	19,863 (46.4)	16,086 (44.3)
Male	13,118 (44.4)	16,801 (45.2)	17,745 (46.3)	22,799 (52.6)	22,985 (53.6)	20,247 (55.7)
Race						
Black	1507 (5.1)	2694 (7.3)	4087 (10.7)	1945 (4.5)	2609 (6.1)	3242 (8.9)
White	26,788 (90.7)	31,656 (85.1)	30,324 (79.0)	38,442 (88.7)	35,214 (82.2)	26,968 (74.2)
API/AN	1249 (4.2)	2826 (7.6)	3960 (10.3)	2978 (6.9)	5025 (11.7)	6123 (16.9)
Surgery						
No	788 (2.7)	1383 (3.7)	3109 (8.1)	1387 (3.2)	1702 (4.0)	3573 (9.8)
Colectomy	28,756 (97.3)	35,793 (96.3)	35,262 (91.9)	41,978 (96.8)	41,146 (96.0)	32,760 (90.2)
Grade						
I	3924 (13.3)	3205 (8.6)	2718 (7.1)	7300 (16.8)	4605 (10.8)	2832 (7.8)
II	13,142 (44.5)	22,465 (60.4)	21,103 (55.0)	20,891 (48.2)	28,276 (66.0)	22,134 (60.9)
IIII	5918 (20.0)	8721 (23.5)	6353 (16.6)	5021 (11.6)	5441 (12.7)	3326 (9.2)
IV	426 (1.4)	371 (1.0)	1084 (2.8)	290 (0.7)	162 (0.4)	501 (1.4)
Unknown	6134 (20.8)	2414 (6.5)	7113 (18.5)	9863 (22.7)	4364 (10.2)	7540 (20.8)
SEER stage						
Localized	9910 (33.5)	13,947 (37.5)	15,895 (41.4)	17,996 (41.5)	19,067 (44.5)	15,329 (42.2)
Regional	13,898 (47.0)	16,572 (44.6)	15,219 (39.7)	17,890 (41.3)	16,609 (38.8)	13,298 (36.6)
Distant	5736 (19.4)	6657 (17.9)	7257 (18.9)	7479 (17.3)	7172 (16.7)	7706 (21.2)
Regional node positivity						
Negative	2374 (8.0)	20,272 (54.5)	20,640 (53.8)	3104 (7.2)	20,835 (48.6)	16,893 (46.5)
<12	1493 (5.1)	12,758 (34.3)	12,416 (32.4)	1733 (4.0)	13,043 (30.4)	11,519 (31.7)
≥12	25,677 (86.9)	4146 (11.2)	5315 (13.9)	38,528 (88.9)	8970 (20.9)	7921 (21.8)

*Note*: Grades: I, well differentiated; II, moderately differentiated; III, poorly differentiated; and IV, undifferentiated or anaplastic.

Abbreviations: AN, Alaska Native; API, Asian/Pacific Islander; IQR, interquartile range; SEER, Surveillance, Epidemiology, and End Results.

### 3.2 Survival analysis

The median CSS for LSCC and RSCC was 19.3 and 16.7 years, respectively, for period A (1975–1989). However, the CSS was not reached during periods B and C—more than half of the cohort was living at the end of the follow‐up period—and thus the median CSS could not be calculated. Median OS increased over time for both LSCC (A = 5.5 years; B = 7.6 years and C = 10.5 years) and RSCC (4.3, 5.8, and 7.5 years respectively).

### 3.3 Cause‐specific survival

The unadjusted CSS sub‐distribution hazard (or hazard ratio [HR] for mortality)^21^ for LSCC versus RSCC was statistically significant for all periods over the study periods (Table 2). The OS over the same time periods were similar, and the strength of relationship for unadjusted mortality grew stronger (Table 2). Adjusted CSS was better for LSCC than RSCC for period C (HR 0.89; 0.86–0.92; *p* < 0.001), but sidedness for periods A and B was not statistically significant. LSCC consistently showed superior OS for all study periods (Table 2). Stage stratification showed worse CSS for localized and regional LCC in the earlier study periods, but the risk attenuated over time (Table 3). However, left sided distant disease had superior CSS for all periods (Table 3).

**TABLE 2:** Trends in cause‐specific survival (CSM) and overall survival (OS) for left‐sided (LSCC) versus right‐sided (RSCC) colon cancer demonstrating unadjusted and adjusted hazard ratios for each of the periods A, B, and C.

Year	CSS^a^				OS^b^			
	HR (95%CI)	*p*‐value*	aHR (95%CI)	*p*‐value*	HR (95%CI)	*p*‐value*	aHR (95%CI)	*p*‐value*
1975–1989	0.94 (0.92–0.96)	<0.001	1.00 (0.98–1.03)	0.831	0.85 (0.84–0.86)	<0.001	0.93 (0.91–0.94)	<0.001
1990–2004	0.92 (0.89–0.94)	<0.001	0.99 (0.96–1.01)	0.293	0.81 (0.80–0.83)	<0.001	0.93 (0.92–0.95)	<0.001
2005–2019	0.91 (0.88–0.93)	<0.001	0.89 (0.86–0.92)	<0.001	0.77 (0.75–0.79)	<0.001	0.84 (0.82–0.85)	<0.001

*Note*: CSS and OS; adjusted for age, race, sex, stage, grade, and surgery.

Abbreviations: aHR, adjusted hazard ratio; CSS, cause‐specific survival; HR, unadjusted hazard ratio.

* *p* < 0.001 for trend, both CSS and OS.

^a^ Fine‐Gray competing risk regression. Adjusted for Other‐Cause specific mortality.

^b^ Cox Proportional hazard regression.

**TABLE 3:** Trends in CSS and OS for left‐sided (LSCC) and right‐sided (RSCC) colon cancer showing adjusted hazard ratios for each of the periods A, B and C and further stratified by tumor stage.

Stage and era of diagnosis	CSS^a^			OS^b^		
	aHR	(95%CI)	*p*‐value	aHR	(95%CI)	*p*‐value
Localized						
1975–1989	1.15	1.09–1.22	<0.001	0.93	0.90–0.95	<0.001
1990–2004	1.06	1.00–1.12	0.058	0.91	0.88–0.93	<0.001
2005–2019	0.95	0.877–1.03	0.211	0.86	0.82–0.90	<0.001
Regional						
1975–1989	1.12	1.08–1.15	<0.001	0.99	0.97–1.02	0.662
1990–2004	1.09	1.05–1.13	<0.001	0.98	0.96–1.01	0.178
2005–2019	0.97	0.92–1.02	0.238	0.88	0.85–0.92	<0.001
Distant						
1975–1989	0.82	0.79–0.85	<0.001	0.79	0.76–0.81	<0.001
1990–2004	0.87	0.84–0.90	<0.001	0.85	0.83–0.88	<0.001
2005–2019	0.82	0.79–0.86	<0.001	0.78	0.75–0.81	<0.001

*Note*: CSS and OS; adjusted for age, race, sex, stage, grade, and surgery.

Abbreviations: aHR, adjusted hazard ratio; CSS, cause‐specific survival; OS, overall survival.

^a^ Fine–Gray competing risk regression. Adjusted for other‐cause specific mortality (OCSM).

^b^ Cox proportional hazard regression.

**FIGURE 2:**
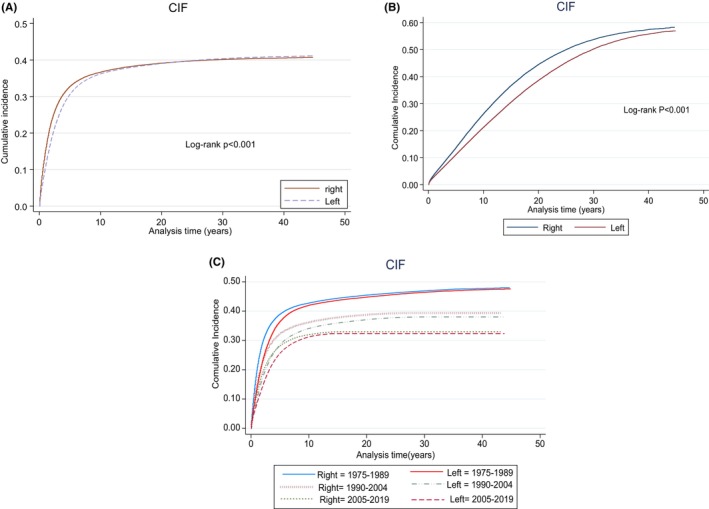
(A) Cumulative incidence function (CIF) graph illustrating the cause‐specific survival (CSS) for right‐ and left‐sided colon cancer spanning 45 years of follow‐up. (B) CIF graph showing other causes of mortality (OCM) for right‐ and left‐sided colon cancer spanning 45 years of follow‐up. (C) CIF graph illustrating the CSS for right‐ and left‐sided colon cancer across various study periods.

**FIGURE 3:**
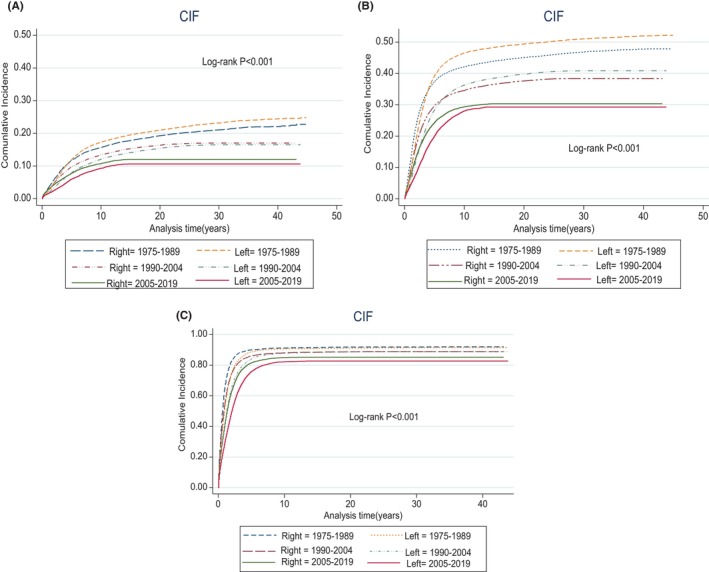
(A) Cumulative incidence function (CIF) graph illustrating cause‐specific survival (CSS) for localized right‐ and left‐sided colon cancer across various study periods. (B) CIF graph showing CSS for regional right‐ and left‐sided colon cancer across various study periods. (C) CIF graph showing CSS for distant right‐ and left‐sided colon cancer across various study periods.

The CIF showed that, irrespective of stage and period of diagnosis, the CSS for RSCC from time of diagnosis until 10 years was worse relative to LSCC (Figure 2A). However, after 20 years of diagnosis, LSCC begins to show worse CSS (*p* < 0.001). We showed that OCM was also superior for LSCC versus RSCC (*p* < 0.001) (Figure 2B). To show the trends in survival over periods A, B, and C, we plotted CIF for CSS for sidedness. RSCC showed worse CSS for all periods versus LSCC. Additionally, CSS for period A (1975–1989) was worse relative to other periods (B, C) (Figure 2C). When sidedness was stratified according to SEER historic staging (Figure 3A–C) and study period, CSS for localized and regional disease was worse for LSCC during 1975–1989. This improved over time.

### 3.4 Overall survival

**FIGURE 4:**
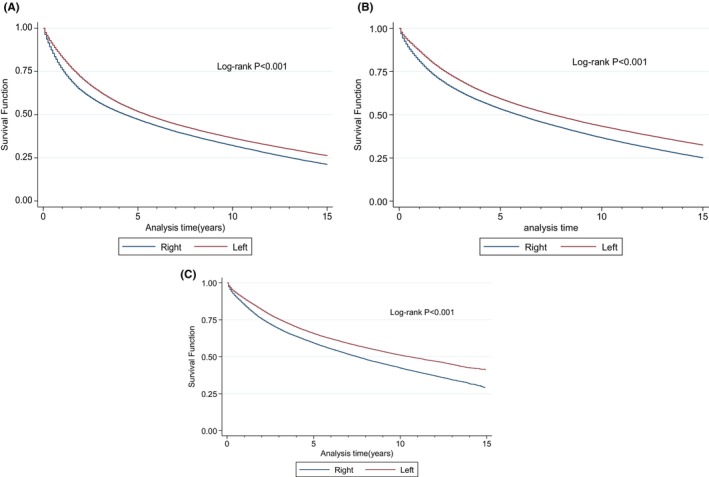
Overall survival (OS) for period A (1975–1989) (A), period B (1990–2004) (B), and period C (2005–2019) (C) for the entire cohort.

**FIGURE 5:**
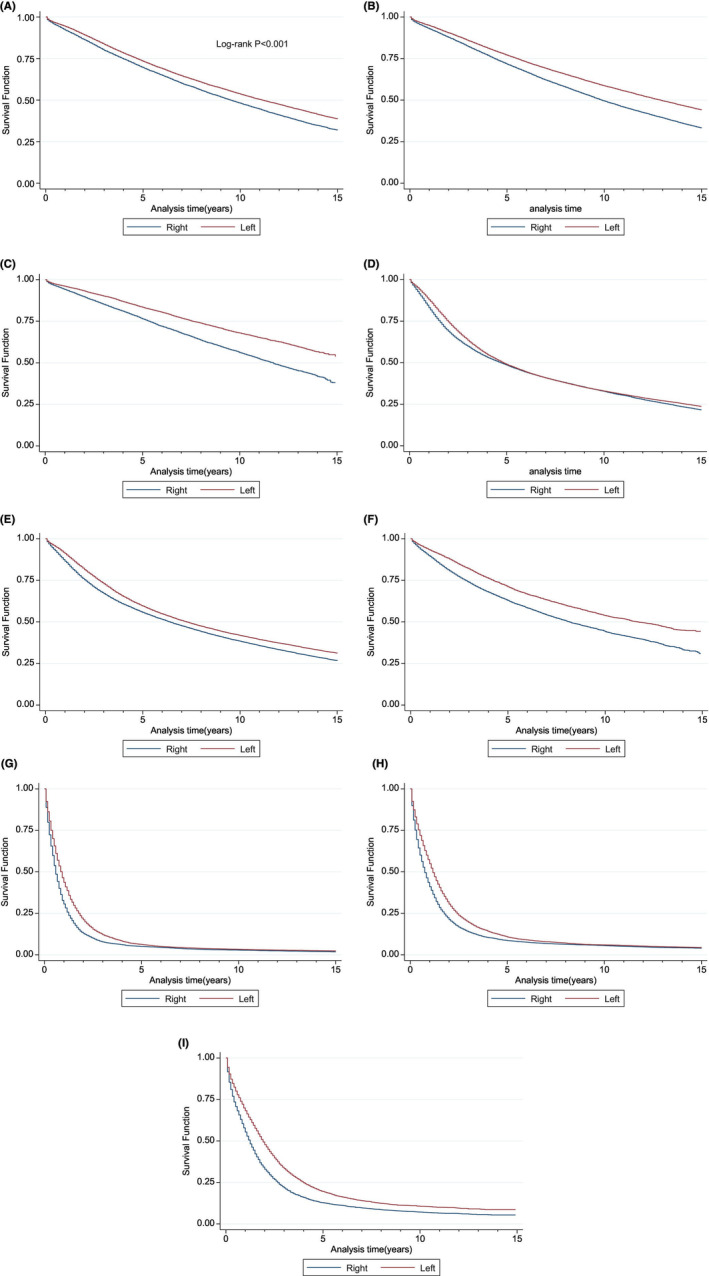
Overall survival (OS) for localized stage for period A (1975–1989) (A), period B (1990–2004) (B), and period C (2005–2019) (C). Overall survival (OS) for regional stage for period A (1975–1989) (D), period B (1990–2004) (E), and period C (2005–2019) (F). Overall survival (OS) for distant disease for period A (1975–1989) (G), period B (1990–2004) (H), and period C (2005–2019) (I).

OS was better for LSCC irrespective of stage, with gradual improvement in adjusted analysis over time (Table 3). The superior OS for LSCC was demonstrated for the entire cohort throughout periods A, B, and C (Figure 4A–C). When stratified by stage, localized disease persistently showed superior OS over the study periods (Figure 5A–C). Regional disease only showed superior OS during the early phase of diagnosis (up until 5 years) during the period A (1975–1989), and the survival advantage disappears after about 5 years of diagnosis (Figure 5D). However, divergence of superior OS in LSCC was evident during period B (1990–2004) and more pronounced in period C (2005–2019) (Figure 5E,F). Divergence of OS for LSCC in the setting of distant disease was also reported for period A (1975–1989), period B (1990–2004), and period C (2005–2019) (Figure 5G–I).

## 4 DISCUSSION

Demographics such as female sex, black race, and older age were associated with an increased frequency of RSCC in our study, while male sex, Asian, Pacific Islander (API)/Alaska Natives (AN) race, and younger age were more likely to be associated with left sided tumors. These findings are similar to other population‐based studies.^7^, ^13^, ^22^ We used competing risk methodology by Fine and Gray^19^ to compete the cause–specific mortality, simultaneously accounting for OCM, which is a significant confounder for cause‐specific survival analysis. LSCC was associated with a superior CSS in the 2005–2019 cohort group compared to RSCC, after adjusting for age, sex, race, historical stage, surgery, and tumor grade. Also, the adjusted OS revealed decreased mortality for LSCC in all cohort groups throughout the 45‐year study period. Other significant findings included inferior CSS for localized LSCC for the 1975–1989 cohort group. In addition, there was a gradual divergence of CSS per SEER stage; distant disease was more pronounced in the later years (period C). The overall shift in survival over the last 45‐year period was significantly more pronounced for LSCC and had increased accentuation over decades for all stages. Our findings had similar patterns to a Norwegian population‐based study and other recent studies.^14^, ^22^

The reason for this observed difference in CSS and OS is likely multifactorial and unlikely to be due solely to the introduction of more effective systemic therapy. This would include various factors associated with the interplay between embryologic development, immunologic function, epigenetic factors, and the microbiome.^23^, ^24^, ^25^ The right colon develops from the embryonic midgut and extends from the cecum to the proximal two‐thirds of the transverse colon, while the left colon develops from the embryonic hindgut and extends from the distal third of the transverse colon to the upper anal canal. The right and left colon are exposed to and have different compositions of intratumoral bacteria that vary along the colorectum. Studies have demonstrated the importance of the microbiome to the development, progression, or prognosis of CRCs.^26^, ^27^, ^28^, ^29^

Molecular mechanisms may account for the differences in CSS for RSCC and LSCC, as microsatellite instability (MSI), BRAF mutations, and RAS mutations have established predictive and prognostic genetic markers for colon cancer.^30^, ^31^ About 15% of all CRCs have mismatch‐repair deficiency (dMMR), and pembrolizumab was shown to have a more favorable prognosis in RSCC than LSCC.^32^ A study suggested the effects of the microbiome affecting the efficacy of programmed cell death protein 1 (PD‐1) blockage.^33^ Several studies have demonstrated MSI to be a favorable independent predictor for colon cancer survival.^31^, ^34^ RSCC predominantly demonstrates MSI and is less common in LSCC, with about 5% prevalence.^35^ A higher proportion of stage II RSCC are MSI positive and associated with a decrease in all‐cause mortality compared to LSCC. However, a lower proportion of stage III right‐sided tumors are MSI positive and associated with increased all‐cause‐mortality compared with LSCC.^13^, ^36^ There are lower MSI positive RSCC in the AJCC stage IV accounting for worse mortality than LSCC.^37^ In addition, there is a significantly decreased risk of distant metastasis as well as lymph node involvement in advanced‐stage disease.^38^

RSCC commonly metastasizes to the peritoneum, leading to peritoneal carcinomatosis, and LSCC more commonly metastasizes to the hepatic and pulmonary systems. There are more options for therapy to slow the progression of the disease than for peritoneal carcinomatosis, which is diffuse on presentation.^7^ Also, a meta‐analysis revealed inferior survival of RSCC with liver metastases treated with surgery or ablation to LSCC.^39^ BRAF and KRAS mutations are common among RSCC^39^, ^40^ which may account for a poorer prognosis.

Advancements in therapeutic agents have significantly affected the prognosis of colon tumor‐sidedness. In the early years, drug therapy for advanced diseases was uncommon. There were no clinical studies on tumor sidedness during the 5‐fluorouracil monotherapy era until the mid‐1990s. Retrospective studies demonstrated hypermutation/hypermethylation in RSCC, causing resistance to 5‐fluorouracil.^30^, ^41^ Rubric et al showed that stage II or III microsatellite stable colon cancers or those with less MSI positivity (more likely to be left) had a better response to 5‐fluorouracil, compared to the colon cancers with high MSI.^30^ This may explain the progressive divergence in survival over our study period, as more and effective chemotherapy, which are favorable to LSCC emerged. Salem et al. found prolonged and improved survival outcome in microsatellite stable, left sided, KRAS and BRAF wild type tumors by adding oxaliplatin to a 5‐fluorouracil backbone.^42^ A meta‐analysis revealed an inferior prognosis of RSCC with chemotherapy with combination chemotherapy and anti‐epidermal growth factor receptor (EGFR) therapy.^43^ In addition to therapies, screening has likely improved colon cancer survival. Population‐based colon cancer screening began in the United States in the late 1980s. Clinical practice guidelines were established in 1997.^44^ It is likely that routine screening with early detection and excision of precancerous polyps has played a significant role in improving outcomes in both LSCC and RSCC. However, it has been established that RSCC is less likely to be detected during routine colonoscopy as compared to LSCC, which may account for disparate outcomes based on the site of the primary neoplasm. The divergence of survival, increasingly tilted towards the LSCC, calls for factoring colon‐cancer sidedness into clinical trials to improve outcomes.

Patients with LSCC demonstrated superior survival throughout all study periods in our analysis, irrespective of stage. However, in a 2015 analysis by Huang et al, authors reported that improved survival in LSCC was significant only among patients with stage III disease.^45^ In addition, at least one other study has suggested that significant survival differences are present only among patients with stage IIIC CRC.^46^ It is unclear why previous studies have demonstrated survival differences only among patients with stage III disease. We hypothesize that our analysis revealed significant survival differences across all stages due to the large sample size, as our cohort included over 200,000 patients whereas previous studies included fewer individuals. However, future large‐scale prospective studies are warranted to assess survival differences between LSCC and RSCC exist across the spectrum of disease.

The superior survival in LSCC relative to RSCC is relatively consistent throughout the medical literature. However, one recent study has called into question the relationship between the primary tumor site and sustained survival. In a 2017 retrospective analysis by Creasy et al, investigators reported that LSCC was associated with improved median OS.^47^ Notably, however, there was no significant association between primary tumor site and long‐term or recurrence‐free survival. The authors hypothesized that the 11‐year follow‐up period allowed for a more thorough assessment of overall patient outcomes. Indeed, although the preponderance of the evidence suggests that RSCC is associated with inferior survival relative to LSCC, further long‐term follow‐up studies similar to those performed by the Creasy group may help establish a more complete understanding of the relationship between colon cancer side and patient outcomes.

Colon cancer side has been shown to represent an important prognosticator in our study and many others. Additional factors that may predict patient outcomes include performance status, baseline carcinoembryonic antigen levels, and number of metastatic sites.^48^ In addition, BRAF mutations have a significant impact on survival, portending a poor prognosis relative to RAS mutations; although BRAF is closely related to primary tumor location, BRAF mutations remain an independent predictor of poor outcomes irrespective of site.^49^

Emerging data suggest that colon cancer sidedness may inform treatment. It has previously been established that LSCC demonstrates increased EGFR expression relative to RSCC, which has important treatment implications in the era of targeted therapies.^50^ Indeed, in a 2015 analysis by Brulé et al, authors found that patients with LSCC demonstrated a superior response to cetuximab—a novel EGFR inhibitor—as compared to those with RSCC.^51^ A similar finding was reported among patients receiving the EGFR inhibitor panitumumab.^52^ In contrast, patients with RSCC tend to respond favorably to immunotherapy, which may be related to the prevalence of dMMR in right‐sided tumors.^53^ Colon cancer sidedness thus appears to have prognostic value not only for survival, but for treatment response.

## 5 LIMITATIONS

This is a retrospective cohort, observational study with potential confounders, such as comorbidities not accounted for. The exclusion criteria were used to reduce the risk of misclassification bias, but consequently may have introduced a degree of selection bias. Molecular markers as surrogate of prognosis were not included in the SEER database and could not be analyzed in our study. The study could not use the AJCC staging to provide more discrete survival picture for various stages and in relation to RSCC versus LSCC. The assignment of primary tumor site using International Classification of Diseases for Oncology (ICDO) varied over the 45‐year period and may result in misclassification, especially since the transverse colon was taken as a single entity rather than based on its embryonic origin. Despite these limitations, this study has added to the emerging evidence to support sidedness as a prognostic factor in colon cancer management.

## 6 CONCLUSIONS

The difference in survival based on colon cancer sidedness predates adoption of newer treatment agents. We have shown that, over the past four decades, LSCC is associated with superior survival over right sided tumors. Furthermore, prognosis between LSCC and RSCC became more divergent in favor of LSCC with the adoption of modern chemotherapy. Clinical trials for colon cancer should consider sidedness when recruiting patients for intervention.

## AUTHOR CONTRIBUTIONS

**Mark B. Ulanja:** Data curation (lead); investigation (lead); methodology (lead); writing – original draft (equal). **Kwabena Oppong Asafo‐Agyei:** Data curation (supporting); investigation (supporting); methodology (supporting). **Vijay Neelam:** Data curation (equal); formal analysis (equal). **Bryce D. Beutler:** Writing – original draft (equal); writing – review and editing (equal). **Daniel Antwi‐Amoabeng:** Investigation (supporting); validation (supporting); writing – review and editing (supporting). **Samuel B. Governor:** Data curation (supporting); formal analysis (supporting); investigation (supporting); methodology (supporting). **Ganiyu A. Rahman:** Supervision (equal); validation (equal). **Francis T. Djankpa:** Supervision (equal); validation (equal); writing – review and editing (supporting). **Reginald N. Ulanja:** Formal analysis (supporting); investigation (supporting). **Grace B. Nteim:** Project administration (equal); writing – review and editing (supporting). **Tarig Mabrouk:** Methodology (equal); writing – review and editing (supporting). **Millicent Amankwah:** Supervision (supporting); validation (supporting). **Olatunji B. Alese:** Supervision (equal).

## Data Availability

All data described in this manuscript are publicly available in the Surveillance, Epidemiology, and End Results (SEER) Program database.
